# Online Detection of Laser Welding Penetration Depth Based on Multi-Sensor Features

**DOI:** 10.3390/ma17071580

**Published:** 2024-03-29

**Authors:** Kun She, Donghui Li, Kaisong Yang, Mingyu Li, Beile Wu, Lijun Yang, Yiming Huang

**Affiliations:** 1 School of Electrical and Information Engineering, Tianjin 300350, China; 2 Tianjin Key Laboratory of Advanced Joining Technology, School of Materials Science and Engineering, Tianjin University, Tianjin 300350, China

**Keywords:** laser welding, spectral analysis, image processing, penetration depth, online monitoring

## Abstract

The accurate online detection of laser welding penetration depth has been a critical problem to which the industry has paid the most attention. Aiming at the laser welding process of TC4 titanium alloy, a multi-sensor monitoring system that obtained the keyhole/molten pool images and laser-induced plasma spectrum was built. The influences of laser power on the keyhole/molten pool morphologies and plasma thermo-mechanical characteristics were investigated. The results showed that there were significant correlations among the variations of the keyhole–molten pool, plasma spectrum, and penetration depth. The image features and spectral features were extracted by image processing and dimension-reduction methods, respectively. Moreover, several penetration depth prediction models based on single-sensor features and multi-sensor features were established. The mean square error of the neural network model built by multi-sensor features was 0.0162, which was smaller than that of the model built by single-sensor features. The established high-precision model provided a theoretical basis for real-time feedback control of the penetration depth in the laser welding process.

## 1. Introduction

Due to the characteristics of high specific strength, good corrosion resistance, and high heat resistance, titanium alloy has been widely used in aerospace, petrochemical, and other fields [1]. Compared to traditional arc welding technologies, laser welding has great advantages in welding titanium alloys owing to high energy density and the small heat-affected zone [2,3]. Since laser welding involves a series of complex processes such as metal melting, solidification, keyhole formation, etc., interference factors in the above processes may affect the welding stability [4]. In addition, the laser-induced plasma affects the interaction between the base material and the laser beam, resulting in the fluctuation in the penetration depth [5]. To acquire high welding quality, it is of great significance to realize the real-time control of the penetration depth.

However, current technical means cannot directly measure the penetration depth in the welding process. Since a variety of physical phenomena are produced and can be detected by sensors, the online prediction of the penetration depth can be realized by establishing the relationship between the sensing information and the penetration depth [6,7]. By Gaussian process regression, Tomcic et al. [8] established a relationship model between laser welding acoustic signals and penetration depth, with a correlation coefficient of up to 87% between the predicted and actual values of the model. Although the acoustic signal is collected conveniently, it is easily affected by environmental noise, which increases the difficulty of data processing. Xiao et al. [9] found that there was a linear relationship between the coaxial infrared radiation temperature and the penetration depth, while it was difficult to determine the emissivity of the weld metal under different temperatures. Using a non-power supply probe, Huang et al. [10] measured the electrical signals of laser-induced plasma and found that the electrical features could be used to indicate weld defects. The plasma electrical signal can be collected at a very high frequency and has a good real-time performance, but it is measured by inserting it into the plasma.

Among several sensing signals, emission spectral signals and optical vision signals have rich information and are widely used in the online monitoring of the laser welding process [11,12,13]. By analyzing emission spectral signals, Mrna et al. proposed that there was a linear relationship between the plasma eruption period and the penetration depth during laser deep penetration welding [14]. Li et al. [15] found that the intensity ratio of specific spectral lines of aluminum and copper was highly correlated with the penetration depth. Furthermore, a prediction model of the penetration depth was established based on a neural network with a prediction accuracy of 0.05 mm. By extracting frequency domain features of plasma and molten pool images, Zhang et al. [16] judged the penetration mode. Using a high-speed camera monitoring system, Liu et al. [17] established the classification model based on the frequency characteristics of the plasma morphology. Liu et al. [18] established a classification model of the weld penetration status with an accuracy of over 98% based on keyhole features. Since a single sensor is susceptible to external interferences, multiple sensors were applied to obtain more comprehensive and accurate welding process information [19,20]. Based on their study of plasma morphology features and the emission spectrum, Li et al. [21] established a prediction model of the penetration state with an accuracy of 97%, much higher than that of a single sensor.

Spectral diagnosis technology can not only analyze the alloy composition and content of welding materials online but also reflect the thermodynamic behavior of laser-induced plasma. However, each spectrogram is composed of a great deal of wavelength-intensity data, so the measured spectral data comprise a huge dataset due to the high sampling rate. How to dig out the features related to the welding process is the primary problem that needs to be solved. At present, several models have been established to predict the penetration state successfully, while the relationship among the plasma emission spectrum, keyhole characteristic, and penetration depths has not been clearly revealed. In addition, the real-time prediction accuracy of penetration depth needs to be improved. In this study, a multi-sensor system was built to acquire plasma spectrum and keyhole/molten pool images during laser welding of titanium alloys. The variation characteristics of spectra and keyhole/molten pool images with welding parameters and penetration depth were explored. Moreover, several models with different sensor features were established and analyzed to acquire an optimal prediction model of the penetration depth. Thus, this article aims to clarify the relationship between multi-sensor features and penetration depth and provide a prediction model of the depth with a high accuracy.

## 2. Experimental Setup and Methods

### 2.1. Experiment System

**Figure 1:**
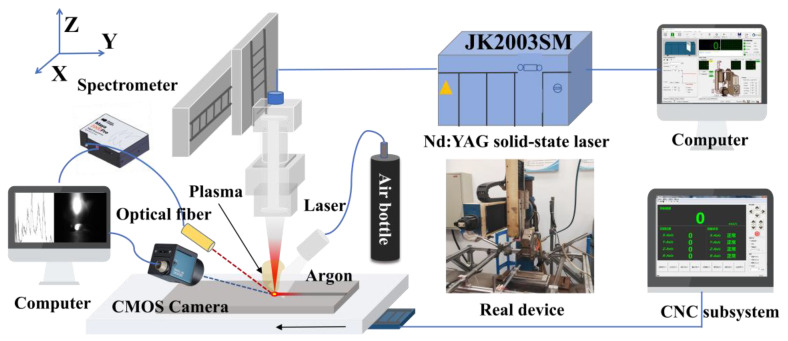
Schematic diagram of the multi-source sensing laser welding monitoring platform.

The laser welding monitoring platform comprises a laser device, CNC motion subsystem, multi-sensor measurement subsystem, and auxiliary equipment, as shown in Figure 1. The JK2003SM Nd: YAG solid-state laser with a wavelength of 1064 nm was adopted. The laser beam transmitted to the laser head through the fiber and focused on the material surface with a diameter of 0.6 mm. The CNC motion subsystem was used to adjust the welding speed. The plasma spectrum was acquired by the spectrometer Ocean Optics, USA, Maya 2000 Pro with a sampling frequency of 50 Hz, of which the spectral range was 350–800 nm, and the resolution was 0.233 nm/pixel. To ensure the collected spectral intensity was within the reasonable measurement range of the spectrometer, a 5% ND filter was used. The laser-induced plasma region is so small that the measurement conditions are harsh. Through repeated adjustments, the measurement angle was set as 11°, and the distance from the melt pool center was 175 mm in order to ensure the acquisition sensitivity and stability of spectral data. The keyhole/molten pool image was acquired by DaHeng, China, MER2-160-227U3M (CMOS) with a maximum frame rate of 227 fps. The auxiliary equipment included the XH-30A split heat-exchange water cooler to cool the laser device and an air pump to protect the laser head. To obtain the clear keyhole/molten pool image, the CMOS camera was equipped with a 650 nm narrow-band filter and a light reducer with a transmittance of 0.3%.

The base material was TC4 titanium alloy, and its chemical composition is shown in Table 1. Before welding, the base material was polished and cleaned to remove the oxide film on the surface of the workpiece. The shielding gas was 99.99% pure argon with a flow rate of 15 L/min. The workpieces moved at a speed of 10 mm/s under the drive of the workbench, and the laser power changed from 800 W to 1300 W in an increment of 100 W.

### 2.2. Plasma Spectrum Processing Method

The spectral data collected during welding included continuous spectrum and linear spectrum. The cubic spline interpolation method was used to process the collected spectral data to remove the influence of continuous spectrum intensity on linear spectrum intensity. Wavelength-intensity measurement is the most direct method to identify elements and contents, but it cannot reflect the thermodynamic properties of the plasma effectively. In this study, the plasma characteristics of the critical transition state from thermal conductive welding to deep penetration welding were deeply investigated by calculating electron temperature and electron density.

#### 2.2.1. Electron Temperature Calculation

For a plasma in local thermodynamic equilibrium (LTE), the excited atoms follow the Boltzmann distribution. The relationship between electron temperature and transition properties is listed as follows [22]:

(1) $$In\frac{I_{ki}\lambda_{ki}}{A_{ki}g_{k}}=-\frac{E_{k}}{kT}+In\frac{N(T)}{U(T)}$$

where $I_{ki}$ is the light intensity of the transition from energy level *k* to *i*, $\lambda_{ki}$ is wavelength, $A_{ki}$ represents the transition probability, $g_{k}$ is the statistical weight of energy level *k*, $E_{k}$ is the excitation energy, *k* is the Boltzmann constant, T is electron temperature, and N(T) and U(T) are total density and partition function, respectively. $In\frac{N(T)}{U(T)}$ is approximately constant. With $E_{k}$ as the abscissa and $In\frac{I_{ki}\lambda_{ki}}{A_{ki}g_{k}}$ as the ordinate, the slope −1/kT can be obtained by linear fitting. Thus, the electron temperature is determined without knowing the total density or partition function. Ti I 499.11 nm, Ti I 500.10 nm, Ti I 500.72 nm, Ti I 506.47 nm, Ti I 519.30 nm, and Ti I 521.04 nm were selected for the calculation of electron temperature. The parameters of these lines are shown in Table 2. Under the parameter of laser power of 1000 W, it was calculated that electron temperature $T_{e}$ = 7659.7 K.

#### 2.2.2. Electron Density Calculation

**Figure 2:**
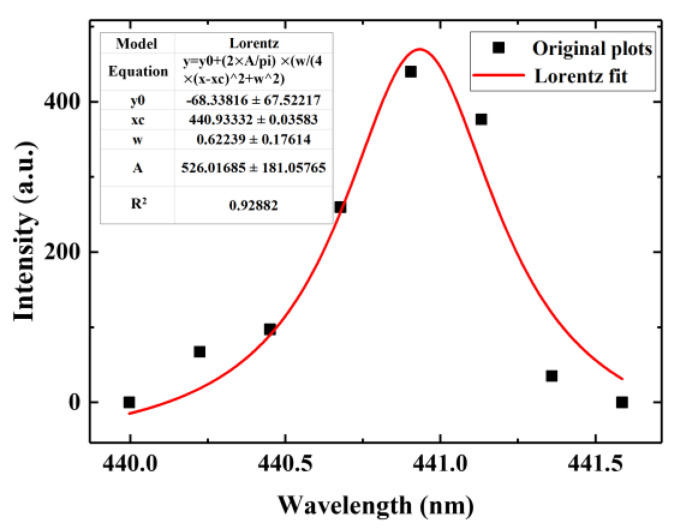
Spectral line broadening of V I 440.82 nm.

The electron density of the plasma can be obtained by measuring the Stark broadening of an isolated atomic spectral line. The important line broadening mechanisms of plasma emission spectra mainly include Doppler broadening $∆{\lambda^{D}}_{FWHM}$ and Stark broadening $∆{\lambda^{S}}_{FWHM}$, caused by the presence of charged electrons and ions. The 440.82 nm V I line was selected for Lorentz fitting to obtain the spectral line broadening contour. The $\Delta \lambda_{1/2}$ is 0.62239 nm, as shown in Figure 2. Doppler broadening can be calculated by Equation (1):

(2) $$∆{\lambda^{D}}_{FWHM}=7.16\times 10^{-6}\times \lambda \times {\left(T/M\right)}^{1/2}\;$$

where λ is the wavelength, T is the temperature, and M is the relative atomic mass. The calculated value is 0.03868 nm. As result, Stark broadening $∆{\lambda^{S}}_{FWHM}$ is 0.58371 nm. Stark broadening $∆{\lambda^{S}}_{FWHM}$ and electron number density $N_{e}$ have the following relationship [24]:

(3) $$∆{\lambda^{S}}_{FWHM}=2\omega \frac{N_{e}}{10^{16}}+3.5A{\left(\frac{N_{e}}{10^{16}}\right)}^{1/4}\times \left(1-\frac{3}{4}N_{D}^{-1/3}\right)\omega \left(\frac{N_{e}}{10^{16}}\right)$$

where ω is the electron collision coefficient, A is the ion broadening coefficient, $N_{e}$ is the electron number density, and $N_{D}$ is the number of particles in the Debye sphere. The first term is the contribution of electron collisions to Stark broadening, and the second term is attributed to ions. For non-hydrogen-like lines, ion collisions contribute little to Stark broadening. Thus, the relationship between the full width at half maxima $∆{\lambda^{S}}_{FWHM}$ and the electrons number density $N_{e}$ can be simplified as follows:

(4) $$∆{\lambda^{S}}_{FWHM}=2\omega \frac{N_{e}}{10^{16}}\;$$

According to the literature [25], $∆{\lambda^{S}}_{FWHM}$ are 2.59 pm and 1.83 pm, corresponding to 5000 K and 10,000 K when $N_{e}=10^{15}{cm}^{-3}$. It was determined that ω is 0.0130 at 5000 K, and ω is 0.0092 at 10,000 K. Since ω is a weak function of temperature, it was calculated that ω is 0.0110 at temperature of 7659.7 K by linear interpolation, and $N_{e}=2.83\times 10^{17}{cm}^{-3}$. In this study, the energy level difference of V I 440.82 nm is about 2.81 eV, and T is 7659.7 K. According to McWhirter criterion, the lowest $N_{e}$ is 3.1 × 10^14^ cm^−3^, which is much lower than the $N_{e}$ obtained by Stark broadening, satisfying the McWhirter criterion and verifying the LTE hypothesis.

### 2.3. Image Feature Extraction

**Figure 3:**
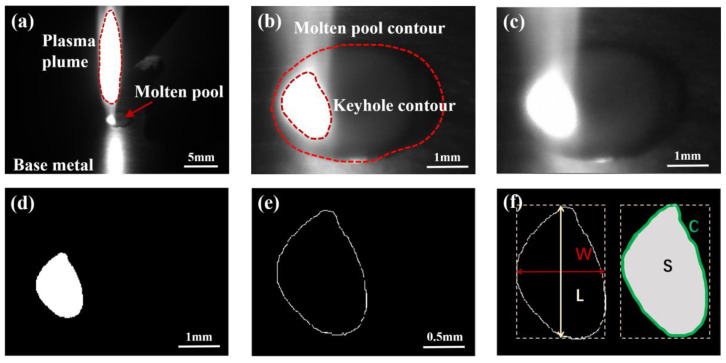
Feature extraction of the keyhole–molten pool: (**a**) an original image; (**b**) region of interest cropping; (**c**) filtering processing; (**d**) binarization processing; (**e**) edge extraction; (**f**) characteristics of the keyhole–molten pool.

The original image taken by a CMOS camera is shown in Figure 3a. Since this study focused on the behavior and morphological characteristics of the keyhole/molten pool, the region of interest, including the entire molten pool, was cropped from the original image, as shown in Figure 3b. The cropped image was filtered and binarized, as shown in Figure 3c,d. The pixel of the keyhole area is 1, and the background pixel value is 0. Then, the Canny operator extracted the keyhole edge contour (Figure 3e). Different welding parameters result in different laser heat inputs, which affect the morphological characteristics of the keyhole and determine the size of the penetration depth. Therefore, there is a specific correlation between the characteristics of the keyhole and the penetration depth. It is seen from Figure 3e that the outline shape of the keyhole is approximately elliptical. Four features are defined: keyhole area S, contour perimeter C, maximum length L, and maximum width W, as shown in Figure 3f.

**Figure 4:**
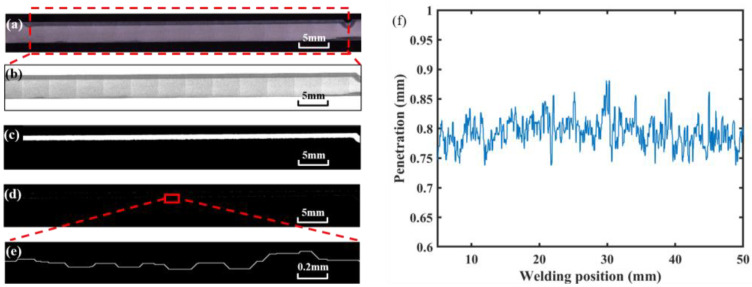
Extraction process of penetration depth: (**a**) original weld longitudinal section; (**b**) interception and gray-scale transformation; (**c**) binarization processing; (**d**) edge extraction; (**e**) an enlarged view of the edge; (**f**) the penetration depth curve.

A method based on image processing was proposed to obtain accurate penetration depth. The specimen was cut along the center of the weld, and the weld section was polished and corroded with Keller reagent, as shown in Figure 4a. First, the picture was cut off to reduce the weld area generated by laser dwell and converted into a gray image (Figure 4b). Secondly, the image binarization was performed, in which the white area represented the weld area, and the black area represented other areas, including the base material, as shown in Figure 4c. Then, the contour of the weld was extracted. Figure 4e is an enlarged view of the red box in Figure 4d. Finally, the weld contour was divided into upper and lower regions according to the left and right intersection points. The difference between the vertical coordinates in the two regions was taken as the penetration depth. The penetration depth curve is shown in Figure 4f. The weld depth of 5 mm at the beginning and 5 mm at the end deviated from the normal value due to the dwell of the laser heat source, so these areas were not considered in the actual analysis.

## 3. Results and Discussion

### 3.1. Influence of Process Parameters on Penetration Depth

**Figure 5:**
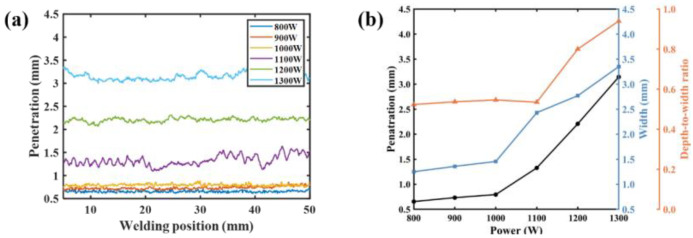
Weld forming results under different laser powers: (**a**) timing variation of penetration; (**b**) average values of penetration depth, weld width and depth/width ratio.

The typical results of molten pool images, plasma spectra, and weld morphologies are shown in Table 3. According to the penetration depth extraction method mentioned above, the time–series variation curves of the penetration depth under different laser powers were obtained, as shown in Figure 5a. It was seen that the penetration depth varied dynamically, even in a single weld. Figure 5b shows the depth/width/depth/width ratio variation curve. It was seen that the weld penetration depth and width increased with the increase in the laser power. The increase in weld width and depth was not a linear process.

When the laser power was 800–1000 W, the weld width and depth were small, and the ratio of depth to width was between 0.52 and 0.55. It was suggested that the heat conduction welding mode worked at this time. When the laser power reached 1100 W, the weld width and penetration depth increased dramatically, and the ratio fluctuated between 0.51 and 0.58. It was in the critical state of heat conduction welding to deep penetration welding, and the unstable weld penetration was presented. When the laser power was 1200–1300 W, the weld width and penetration depth increased rapidly with the increase in laser power. It was the laser deep penetration welding mode, and the ratio of depth to width was between 0.8 to 0.94. It is well known that the penetration depth depends on the amount of laser energy absorbed. The absorption of laser energy mainly depends on the combined action of the dissipation by metal vapor/plasma and the absorption in the keyhole. Under the above combined action, the absorption rate of laser energy shows a sharp increasing trend with the transition from heat conduction welding to deep penetration welding, resulting in the penetration depth increasing sharply.

### 3.2. Plasma Spectral Analysis

**Figure 6:**
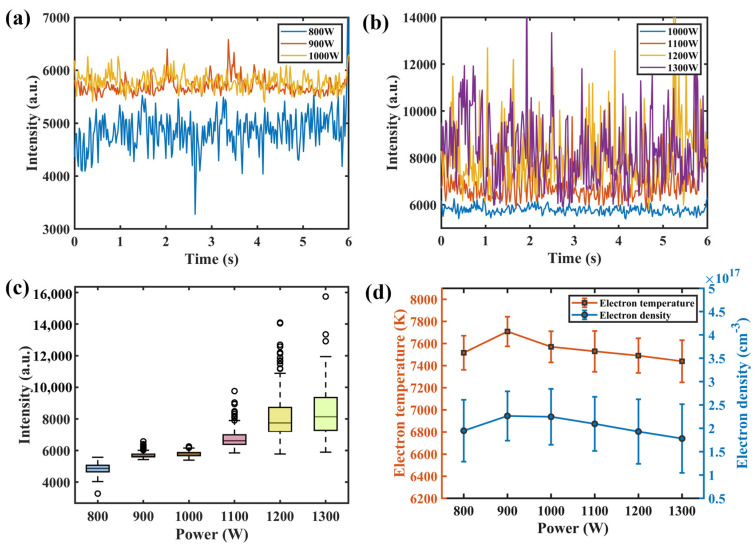
Variation of plasma spectral intensity and plasma thermomechanical parameters under different parameters: (**a**) emission spectral intensity of Ti I 506.47 nm under laser power of 800–1000 W); (**b**) 1000–1300 W; (**c**) box diagram of spectral intensity of Ti I 506.47 nm; (**d**) error bar graphs of plasma electron temperature and density.

The time–series variation curves of the relative intensity of the spectral line Ti I 506.47 nm under different parameters are shown in Figure 6a,b. It was seen that the emission spectral intensity changed dynamically during the welding process. Figure 6c shows the box diagram of the spectral intensity of Ti I 506.47 nm. As a whole, the spectral intensity of laser-induced plasma increased with the increase in laser power. However, the difference in spectral intensity between laser power at 900 W and 1000 W was slight. When the laser power reached 1100 W, the value and fluctuation in spectral intensity increased significantly. Figure 6d shows the error bar diagram of laser-induced plasma electron temperature and electron density under different parameters. The variation trend of plasma electron density was the same as electron temperature. The plasma thermomechanical parameters increased first and then decreased with the increase in laser power, which was not wholly consistent with the variation trend of spectral intensity. At a laser power of 1100 W, the plasma electron temperature and density fluctuated wildly.

**Figure 7:**
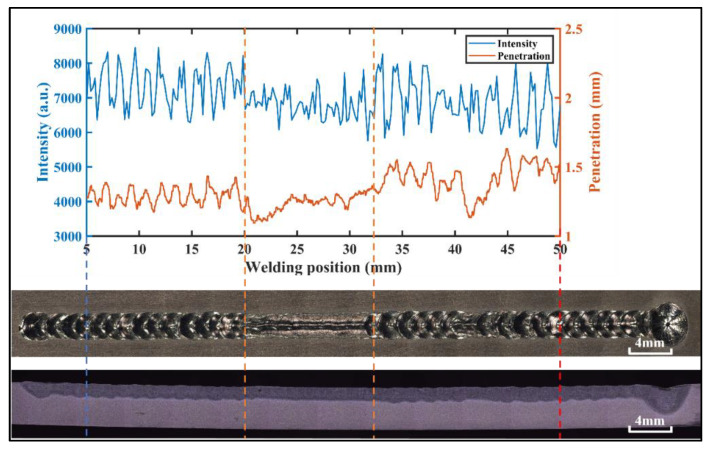
Corresponding figure of spectral intensity—penetration depth—weld surface morphology—weld longitudinal section.

Figure 7 shows the corresponding diagram of spectral intensity—penetration depth—weld surface morphology—weld longitudinal section when the laser power is 1100 W in an experiment. When the laser power was 1100 W, it was found that the spectrum fluctuation was consistent with the weld surface forming and penetration depth fluctuating.

### 3.3. Keyhole–Molten Pool Images Analysis

**Figure 8:**
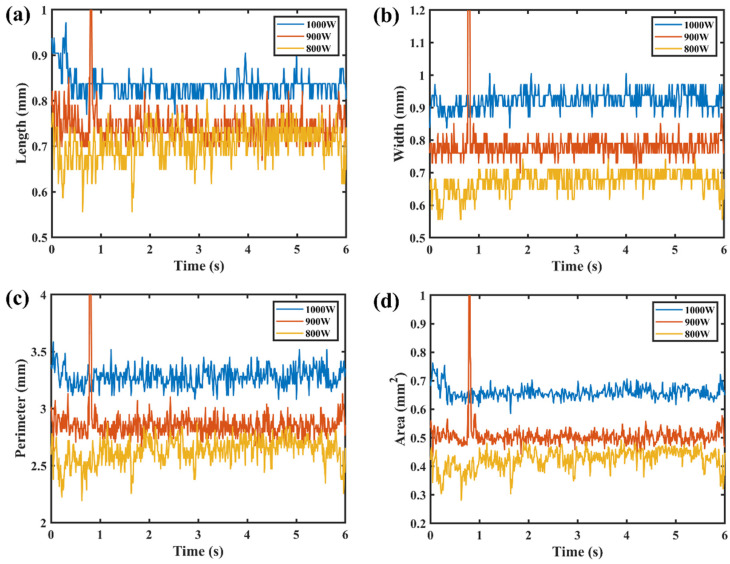
Sequence diagram of the image features of the keyhole–molten pool at 800–1000 W laser power: (**a**) length; (**b**) width; (**c**) perimeter; (**d**) area.

**Figure 9:**
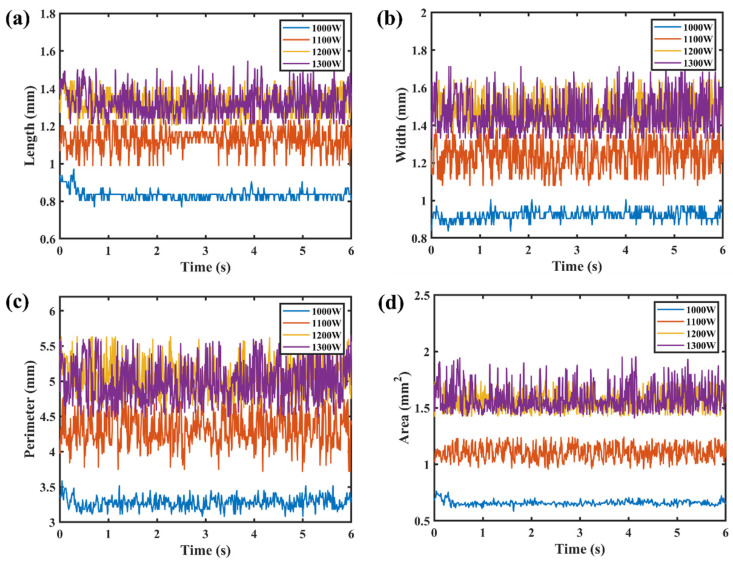
Sequence diagram of keyhole–molten pool image features at 1000–1300 W laser power: (**a**) length; (**b**) width; (**c**) perimeter; (**d**) area.

Figure 8 and Figure 9 show the time series of image features at different laser powers. During the welding process, the keyhole–molten pool area was mainly affected by the ablation pressure, the surface tension, and the ablation pressure [26]. It was proposed that ablation pressure was the key driving force for the formation of the keyhole, and it had a nonlinear, positive correlation with the material surface temperature [27,28]. With the increase in laser power, the surface temperature and ablation pressure of the material increased, and the image features showed a nonlinear increase trend. When the laser power was less than 1100 W, it was the heat conduction welding mode. The ablative pressure and the surface tension of the molten pool were slight, and the fluctuation in the image features of the keyhole–molten pool was slight. The critical state for the transition from heat conduction welding to deep penetration welding is when the laser power is 1100 W. The ablative pressure was sufficient to form a keyhole, the surface tension of the molten pool increased, and the features of the keyhole–molten pool showed a significant fluctuation. When the laser power was greater than 1100 W, the fluctuation in the keyhole–molten pool image features increased further.

### 3.4. Establishment of Penetration Prediction Models

As mentioned above, the weld penetration depth was consistent with the plasma spectral intensity and keyhole/molten pool image features in the heat conduction welding mode with laser power of 800–1000 W. Therefore, sensor features could predict the weld penetration depth.

**Figure 10:**
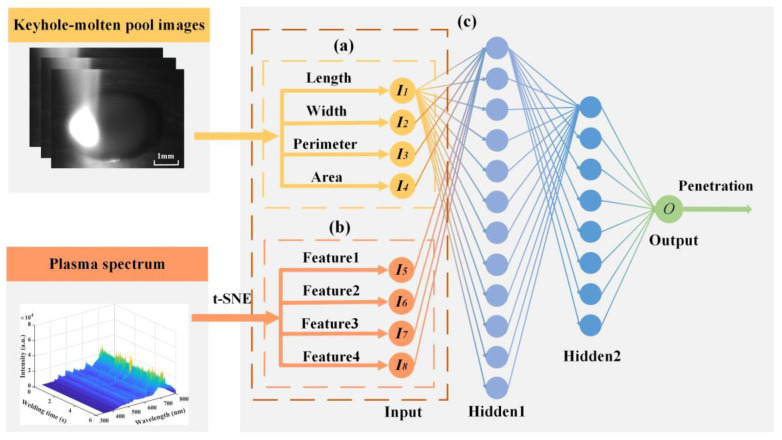
Structure of BP neural network: (**a**) BP neural network based on keyhole–molten pool images; (**b**) BP neural network based on plasma spectrum; (**c**) BP neural network based on multi-sensor features.

BP (back propagation) neural network constructed the mapping relationship between the input features and the output results, which was infinitely approximated to any continuous function in the closed interval. In this paper, a BP neural network containing two hidden layers was constructed to predict weld penetration depth, as shown in Figure 10. Three models were constructed by selecting single- or multiple-sensor features of the stable welding stage as inputs and weld penetration depths as outputs. In total, 80% of the data was selected as the training set and 20% as the testing set. Three indexes, namely R^2^ (R-squared), RMSE (root mean square error), and MAE (mean absolute error), were used to evaluate the accuracy of each model.

**Figure 11:**
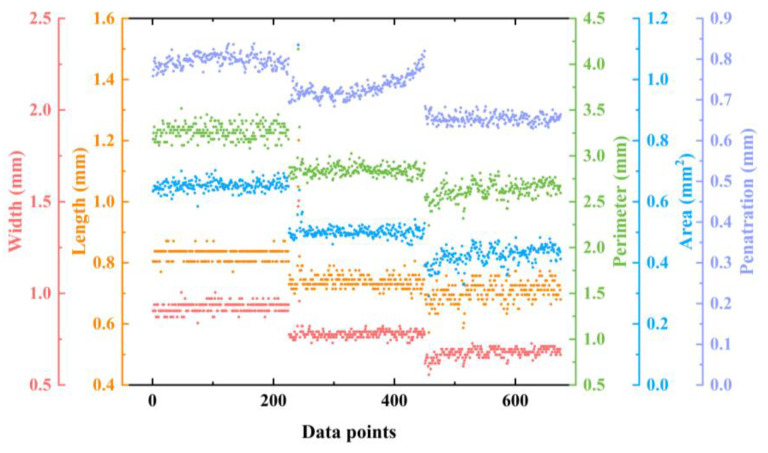
Features and labels visualization of keyhole/molten pool images.

**Figure 12:**
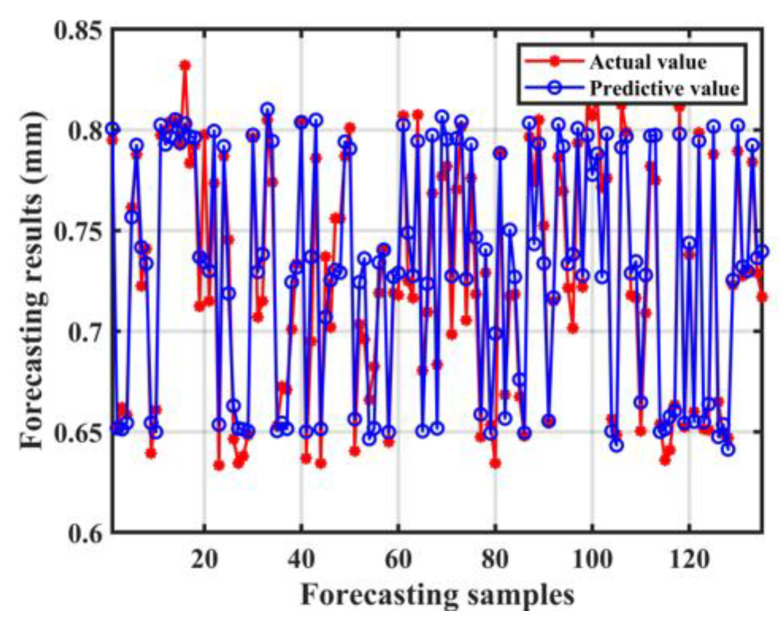
The prediction results of the model trained by keyhole/molten pool images.

With length, width, perimeter, and area as input and corresponding weld penetration depth as output, the prediction model of penetration depth based on visual sensor signal was established. Figure 11 shows the visual features and labels used for training the model. The prediction results of the testing set are shown in Figure 12. The R^2^ (R-squared) of the model was 0.8966, RMSE was 0.0189, and MAE was 0.0149. The results showed a good fit between the real penetration depth and the predicted value, and the error was small.

**Figure 13:**
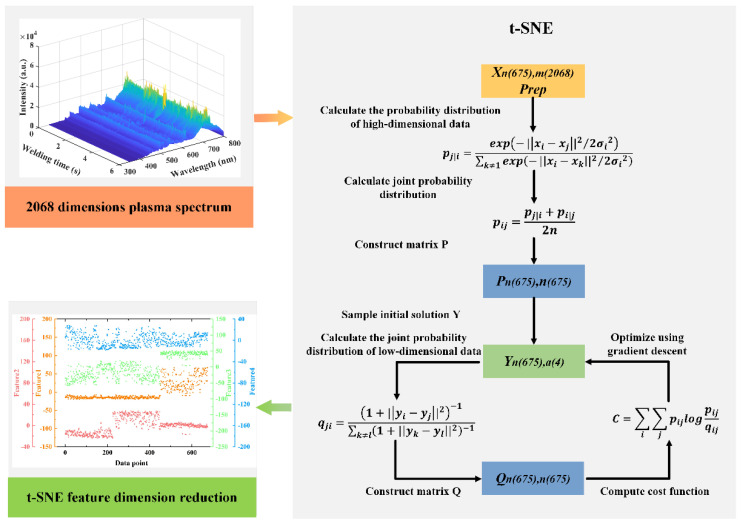
t-SNE dimension reduction flow chart of plasma spectral features.

Each spectrum covered 2068 wavelength dimensions, including ultraviolet, visible, and infrared. The rich spectral information was conducive to understanding the welding process. However, finding the relationship between spectral data and weld penetration was challenging due to information redundancy. Therefore, it was necessary to reduce the data dimensions before building the prediction model. The data of 2068 dimensions were reduced to four dimensions by t-SNE, as shown in Figure 13. With these four dimensions as the input of the model and the penetration depth as the output of the model, the penetration depth prediction model was established. The R-squared of the model was 0.9052, RMSE was 0.0194, and MAE was 0.0146.

**Figure 14:**
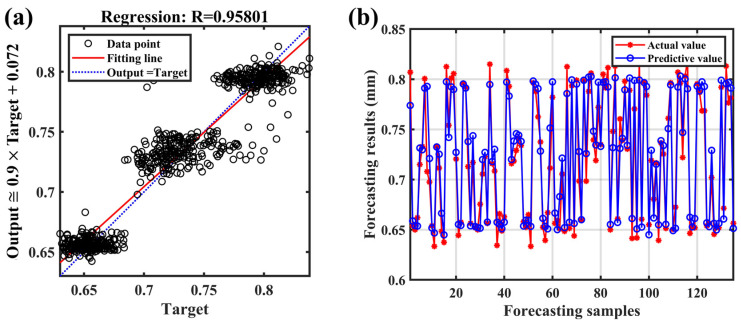
The model trained by on multi-sensor features: (**a**) linear regression diagram; (**b**) prediction results.

**Figure 15:**
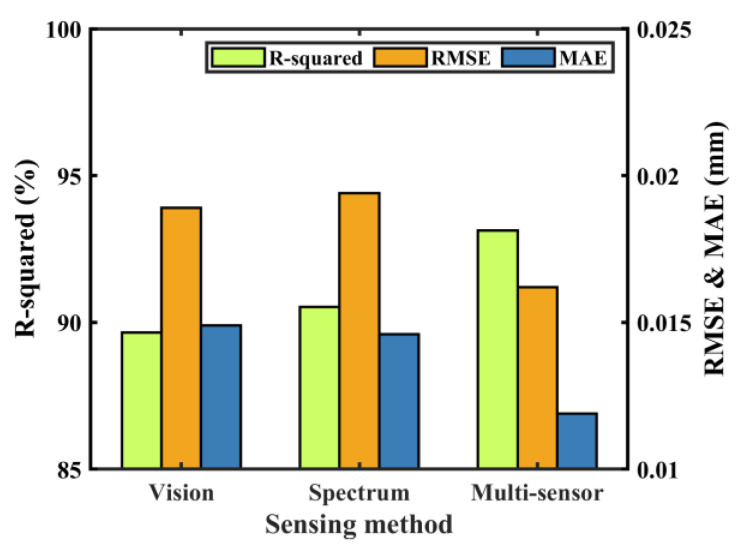
Evaluation indicators of the three models.

A penetration prediction model was established with four spectral features, four image features as inputs, and the corresponding weld penetration values as outputs. The model trained by multi-sensor signals is shown in Figure 14. The R-squared of the model was 0.9313, RMSE was 0.0162, and MAE was 0.0119. The prediction results of the above models were compared, as shown in Figure 15. It was seen from the evaluation index that the fit degree trained with multi-sensor features was significantly improved compared with the model trained by single-sensor features. The difference between the predicted and observed penetration values was also reduced. Measuring various associated information generated in the laser welding process by optical sensing is an effective means to understand the welding process and predict the penetration depth. However, each sensing method has its limitations, and there may be errors in predicting the penetration depth. Combining a variety of optical measurement methods, the prediction accuracy of penetration depths can be improved.

## 4. Conclusions

1. The thermomechanical characteristics of titanium alloy laser-induced plasma under different laser powers were studied. The time series of electron temperature and density during titanium alloy laser welding were calculated by using 6 Ti I spectral lines and 1 VI spectral line. When the laser power increased from 800 W to 1300 W, the average electron temperature increased from 7516.1 K to 7708.3 K and then decreased to 7438.9 K. The average electron density increased from 1.94 × 10^17^ cm^−3^ to 2.26 × 10^17^ cm^−3^ and then decreased to 1.78 × 10^17^ cm^−3^;
2. The penetration depth, the spectral intensity of Ti I 506.47 nm, and four features of the keyhole–molten pool images were obtained by feature extraction. The feature values showed the same change trend with the increase in laser power. The penetration depth increased from 0.65 mm to 3.14 mm, and the spectral intensity of Ti I 506.47 nm changed from 4860 a.u. to 8363 a.u. The keyhole/molten pool area increased from 0.42 mm^2^ to 1.62 mm^2^;
3. The sensor features and weld penetration depth had a positive correlation. Two BP neural network prediction models of penetration depth based on single-sensor features were established. The correlation coefficients between the four image features and penetration depth were larger than 0.768. The R^2^, RMSE, and MAE of the neural network model established by image features were 0.8966, 0.0189, and 0.0149, respectively. As for spectral data, with four features obtained by t-SNE dimensionality reduction as inputs, the R^2^, RMSE, and MAE are 0.9052, 0.0194, and 0.0146, respectively;
4. A BP neural network prediction model of penetration depth was established by using the four image features and the four spectral features after dimensionality reduction as inputs. The R^2^ was 0.9313, and the RMSE was 0.0162, which meant the fitting degree was higher, and the error was smaller than those of the BP neural network model trained by single-sensor features;
5. In this study, the relationship between sensing features and penetration depth was investigated. A high-precision penetration depth prediction model was established. On the basis of the above model, an online detection software system of the penetration depth will be built through open-source language as well as a control model of the penetration depth.

## Figures and Tables

**Table 1:** Chemical composition of TC4 titanium alloy used.

Element	Ti	Al	V	Fe	O	C	N	H
Weight (%)	Balance	5.5~6.8	3.5~4.5	≤0.30	≤0.20	≤0.10	≤0.05	≤0.015

**Table 2:** Parameters used for calculating electron temperature [23].

Line	*Λ* (nm)	Transitions	Excitation Energy (eV)		*g_k_*	*A_ki_* (s^−1^)
			*E_k_*	*E_i_*		
Ti I	499.11	3d3F44pyG5°5→3d3F44sa5F4	3.3194250	0.8359952	11	5.84 × 10^7^
Ti I	500.10	3d24sF45seF35→3d2F34s4pP3zG4°5	4.4754008	1.9968989	7	3.65 × 10^7^
Ti I	500.72	3d3F44saF25→3d3F44pyG3°5	3.2935658	0.8181426	7	4.92 × 10^7^
Ti I	506.47	3d2F34s4pP°3zD3°3→3d24s2aF43	2.4953137	0.0479663	7	4.37 × 10^6^
Ti I	519.30	3d2F34s4pP°3zF3°3→3d24s2aF33	2.4079690	0.0210938	7	3.86 × 10^6^
Ti I	521.04	3d2F34s4pP°3zF4°3→3d24s2aF43	2.4268635	0.0479663	9	3.89 × 10^6^
Ti II	430.00	3d2F34pzD7/2°4→3d3aP5/24	4.0626145	1.1801004	8	1.63 × 10^7^
V I	440.82	3d4D54pyF5/2°6→3d4D54saD5/26	3.0870583	0.2752643	6	5.1 × 10^7^

**Table 3:** Optoelectronic signal and weld morphology at different laser power (with the welding speed of 10 mm/s).

Laser Power (W)	Keyhole–Molten Pool Images	Plasma Spectrum	Weld Morphologies
800	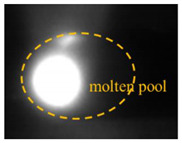	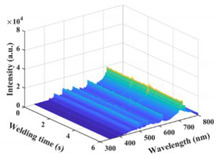	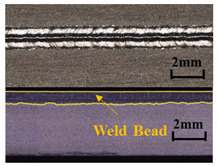
1100	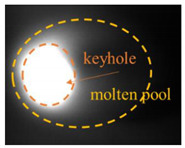	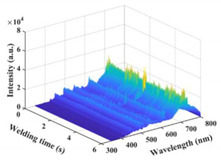	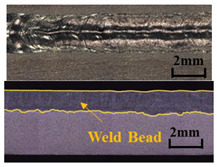
1200	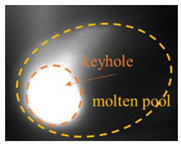	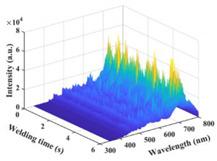	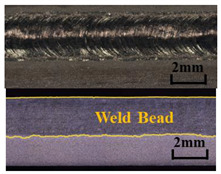

## Data Availability

Data are contained within the article.
